# Acknowledgement to Reviewers of *Metabolites* in 2018

**DOI:** 10.3390/metabo9010009

**Published:** 2019-01-08

**Authors:** Metabolites Editorial Office

**Affiliations:** MDPI, St. Alban-Anlage 66, 4052 Basel, Switzerland

Rigorous peer-review is the corner-stone of high-quality academic publishing. The editorial team greatly appreciates the reviewers who contributed their knowledge and expertise to the journal’s editorial process over the past 12 months. In 2018, a total of 95 papers were published in the journal, with a median time to first decision of 17 days and a median time to publication of 38 days. The editors would like to express their sincere gratitude to the following reviewers for their cooperation and dedication in 2018:

Adam, TomášLi, YongAfshinnia, FarsadLin, Chung-HoAlaimo, SalvatoreLiu, XiaojingAllen, Doug K.Liu, Yen-NienAmathieu, RolandLiu, YouzhongAmato, JussaraLudwig, ChristianAnand, SwatiMa, TengAraujo, PedroMackinder, LukeArias-Borrego, AnaMadsen, JonnaAriza, Maria EugeniaMangalagiu, IonelArtati, AnnaMarczin, NandorArthur, ArthurMarincola, Flaminia CesareBackman, TylerMarkley, JohnBaloni, PriyankaMármol, InesBanfalvi, ZsofiaMarrubini, GiorgioBarlow, ChristopherMartí, RaúlBarupal, DineshMartin, Francois-PierreBasetti, MadhuMaruyama, KeiBermingham, EmmaMathé, Ewy A.Bernacchioni, CaterinaMatijašic, MarioBertram, Hanne ChristineMatsuda, FumioBiales, AdamMcConville, MalcolmBingol, KeremMedana, ClaudioBirkemeyer, ClaudiaMendoza-Cozatl, DavidBišová, KateřinaMetallo, ChristianBoard, MaryMielcarek, MichalBojko, BarbaraMikulic-Petkovsek, MajaBoros, LaszloMillet, OscarBrantley, Milam A.Misra, BiswapriyaBrebu, MihaiMlynarz, PiotrBroeckling, CoreyMomken, ImanBruce, ClintonMonge, María EugeniaBruinsma, BoteMor, MarcoBuckel, WolfgangMori, MatteoBurger, MichaelMuehlbauer, Michael J.Burhans, MaggieNadal-Desbarats, LydieCalingacion, MariafeNagayama, YujiCampos, BrunoNakayama, YasumuneCapozzi, FrancescoNaponelli, ValeriaCappello, TizianaNaviaux, Robert K.Caricofi, Aulus CavalieriNotarnicola, MariaCarqueijeiro, InêsObanda, DianaCarradori, SimoneObermüller, NicholasCarter, ClaireOh, Min KyuCarvalho Carmona, Maria JoséOikawa, AkiraCasabianca, LeahOse, JenniferCheca, AntonioOvermyer, KatherineChen, ChiPaglia, GiuseppeChoudhary, Kumari SonalPastor, VictoriaCiaccio, MarcelloPeffers, MandyCiaramelli, CarlottaPereira, AnaCiborowski, MichalPešić, MilicaConte, PellegrinoPetkova, NadezhdaCorbet, CyrilPhan Nguyen, AnCordaro, MarikaPidatala, Venkata RamanaCox, JamesPiepenbrink, KurtCsanaky, Iván L.Pinkas, AdiCuperlovic-Culf, MiroslavaPinu, Farhana R.Cwiklik, LukaszPiva, TerrenceDanezis, GeorgiosPlaza, AlbertoDe La Fuente, Macarena I.Plaza-Díaz, JulioDe Santo, CarmelaPowers, RobertDe Simone, GiuseppinaPozo, Óscar J.Del Coco, LauraPriefer, RonnyDolinsky, Vernon W.Prieto Vidal, NataliaDomínguez-Álvarez, EnriquePromislow, DanielDunn, WarwickQuintana, Fancisco JavierEisenreich, WolfgangRaftery, DanielEnjalbert, FrancisRagusa, AndreaErnst, MadeleineRamos-Suárez, Juan LuisEvans, CharlesRana, SatianderEverett, JeremyRango, MarioFabris, MicheleRaynaud, FlorenceFærgeman, NilsRogers, SimonFasulo, SalvatoreRoscini, LucaFedorova, Olga V.Ross, IanFenaille, FrancoisRotondo, ArchimedeFendt, Sarah-MariaRöttger, RichardFiandaca, MassimoRubert, JosepFukuda, TomohikoRupasinghe, ThusithaGalla, Hans-JoachimSailasuta, NapaponGarbayo, InésSalanta, LianaGarcia Martin, HectorSalek, RezaGarcía-Barrera, TamaraŠamec, DunjaGarrett, TimothySanchez, LauraGaul, DavidSandhu, AmanGenta-Jouve, GrégorySandín-España, PilarGhesquiere, BartSandusky, Peter OlafGill, Pritmohinder S.Sas, KelliGillies, RobertSchirra, HorstGiskeødegård, Guro F.Schnackenberg, LauraGoldoni, LucaSchymanski, EmmaGolovko, MikhailŚcibisz, IwonaGonda, SándorSeino, ManabuGonzález-Domínguez, RaúlSekula, PeggyGoudarzi, MaryamSeltzman, Herbert H.Gowda, G. A.Sengupta, ArjunGraham, StewartSerkova, NatalieGramsbergen, Jan BertSharma, DipaliGrant, David F.Shaw, WilliamGrasso, GiuseppeShoaie, SaeedGreen, BrianSilva, Margarida F. B.Greenlief, MichaelSimirgiotis, MarioGregory, StephenSimón-Manso, YamilGromova, MarinaSimonsen, Anja HviidGrösch, SabineSingh, ChandraGu, HaiweiSmilde, AgeGuido, Luis F.Smith, AlexanderGuo, YinSnowden, StuartHaarmann-Stemmann, ThomasSterken, MarkHalama, AnnaStringer, Kathleen A.Halldórsson, SkarphéðinnSubramanian, ChitraHan, JaehongSulek, KarolinaHanhineva, KatiSvastova, EliskaHart, Sarah R.Takami, TaroHarvey, Patricia J.Tapio, IlmaHashimoto, MakotoTheodoridis, GeorgiosHassan, Sammer-UlTimperio, Anna MariaHaudenschild, Dominik R.Tolstikov, VladimirHeaney, LiamTourniaire, FranckHeinzmann, Silke SophieToya, YoshihiroHibi, TakaoTrainor, PatrickHoene, MiriamTsaltas, DimitriosHolt, Scott M.Turano, PaolaHuan, TianxiouVan Gastelen, SanneHumer, ElkeVan Hasselt, CoenHyötyläinen, TuuliaVentura, NatasciaIlies, Marc A.Wähälä, KristiinaIorio, EgidioWahi, KanuIslam, M. NurulWahli, WalterIvanisevic, JulijanaWang, KunJaumot, JoaquimWang, LeiJeong, Tae-YongWang, PengchengJouret, FrançoisWang, XinwenKabil, OmerWang, ZenengKamikawa, RyomaWase, NishikantKamlage, BeateWatson, DavidKato, YasumasaWeighardt, HeikeKelly, Rachel S.Weljie, AalimKhachik, FredWhiteson, KatrineKhadka, ManojWichert, BrigittaKim, In-JungWilliams, Noelle S.Kim, Young-MoWilson, ThomasKind, TobiasWińska, KatarzynaKo, DennisWissenbach, Dirk K.Kremling, AndreasWitting, MichaelKubo, AtsushiWong, AlanKus, PiotrWong, Ka-ChunLahiri, TanayaWu, SiLalk, MichaelXiong, WeiLavoie, PascalYamamoto, SuguruLe Borgne, MarcYoneyama, TadakatsuLerche, Mathilde H.Zalubovskis, RaivisLi, Fu-shuangZhang, BoLi, KefengZhang, KaiLi, ShuzhaoZhou, LeiLi, YanZhu, ChangfuLi, YinZierer, Jonas

